# Hypertrophy of Ligamentum Flavum in Lumbar Spine Stenosis Is Associated with Increased miR-155 Level

**DOI:** 10.1155/2014/786543

**Published:** 2014-05-18

**Authors:** Jianwei Chen, Zude Liu, Guibin Zhong, Lie Qian, Zhanchun Li, Zhiguang Qiao, Bin Chen, Hantao Wang

**Affiliations:** ^1^Department of Orthopedics, Renji Hospital, School of Medicine, Shanghai Jiaotong University, 1630 Dongfang Road, Shanghai 200127, China; ^2^Shanghai Key Laboratory of Orthopaedic Implants, Department of Orthopaedics, Ninth People's Hospital, Shanghai Jiaotong University School of Medicine, 639 Zhizaoju Road, Shanghai 200011, China

## Abstract

Hypertrophy of ligamentum flavum (LF) contributes to lumbar spinal stenosis (LSS) and is caused mainly by fibrosis. Recent data indicate that miR-155 plays a crucial role in the pathogenesis of different fibrotic diseases. This study aimed to test the hypothesis that miR-155 exerts effects on LF thickness by regulating collagen expression. We found that LF thickness and the expression of collagen I and, collagen III were higher in LF from LSS patients than in LF from lumbar disc herniation (LDH) patients (*P* < 0.01). The expression of miR-155 was significantly higher in LF from LSS group than in LF from LDH group (*P* < 0.01). miR-155 level was positively correlated with LF thickness (*r* = 0.958, *P* < 0.01), type I collagen level (*r* = 0.825, *P* < 0.01), and type III collagen level (*r* = 0.827, *P* < 0.01). miR-155 mimic increased mRNA and protein expression of collagen I and collagen III in fibroblasts isolated from LF, while miR-155 sponge decreased mRNA and protein expression of collagen I and III in fibroblasts. In conclusions, miR-155 is a fibrosis-associated miRNA and may play important role in the pathogenesis of LF hypertrophy.

## 1. Introduction

Lumbar spinal stenosis (LSS) is a common condition in elderly patients. LSS is defined as the narrowing of the spinal canal with cord or nerve root impingement which results in the symptoms of radiculopathy or pseudoclaudication [1]. Hypertrophy of the ligamentum flavum (LF) is usually involved in the pathogenesis of LSS, which can reduce the diameter of the spinal canal and compress the dural sac and nerve roots, resulting in symptoms, even in the absence of a bulging annulus fibrosus or herniated nucleus pulposus or osseous spurs [2–4].

LF is a well-defined elastic structure that consists of elastic (80%) and collagen (20%) fibers [5]. Hypertrophied LF tissues become disorganized and show decreased levels and degeneration of elastic fibers but increased levels of collagen fibers [6, 7]. During LF hypertrophy, there are increases in the expression and activity of various molecules, including matrix metalloproteases (MMPs) [8–10], tissue inhibitors of matrix metalloproteases (TIMPs) [11], platelet-derived growth factor-BB (PDGF-BB) [12], connective tissue growth factor (CTGF) [13], bone morphogenetic protein (BMP) [14], and inflammatory cytokines [15–17].

microRNAs (miRNAs) are evolutionarily conserved, single-stranded noncoding RNA molecules of 19–24 nucleotides that control gene expression at the posttranscriptional level. miRNA expression signatures have been associated with clinicopathological features and the outcomes of different diseases [18]. However, very little is known about the role of miRNAs in LF hypertrophy. miRNAs play a crucial role in tissue degradation and fibrosis [19–21]. miRNAs could promote cartilage degradation through regulating the expression of genes encoding catabolic factors such as MMP and ADAMTS [22, 23]. Notably, a significant increase in the expression of miR-155 was observed in fibroblast cells and tissues from rheumatoid arthritis patients [24]. MiR-155 is a typical multifunctional miRNA that plays crucial role in various physiological and pathological processes, such as hematopoietic lineage differentiation, immunity, cancer, cardiovascular disease, and inflammatory response [25, 26].

In this study, we aimed to explore the potential role of miR-155 in the development of LF hypertrophy in patients with LSS. We compared the thickness, elastin degradation, fibrosis, collagen I and collagen III expression, and miR-155 expression in LF from patients with LSS to those from patients with lumbar disc herniation (LDH). Next, we investigated the correlation between miR-155 level and LF features. Finally, we examined the effects of miR-155 on the expression of types I and III collagen in cultured human LF cells.

## 2. Methods

### 2.1. Specimens

LF samples were obtained from 15 patients (7 male, 8 female, average age: 65.67 years old, range: 63–71 years) who underwent decompressive laminectomy due to symptomatic degenerative lumber spinal stenosis. As the control, LF samples were obtained from 15 patients (10 male, 5 female, average age: 25.17 years old, range: 20–30 years) with lumbar disc herniation who were operatively managed for this disorder. The LF were sampled from L4/5 and then subjected to histological staining, Masson's trichrome staining, immunohistochemical analysis, and biological evaluation. The study was approved by the institutional ethics review board with written informed consent obtained from each patient.

### 2.2. LF Thickness Measurement

Magnetic resonance imaging (MRI) was performed to measure the thickness of the LF in each of the 30 patients. On the axial T1-weighted image through the facet joint, the LF was clearly observed as a low-signal intensity mass just at the ventral side of the facet joint [27]. The maximum thickness of the LF was traced using the manual cursor technique by an experienced surgeon and measured automatically in the PACS system. The measurement of each ligament was repeated three times, and the average value was used as the final thickness of LF.

### 2.3. Histological Analysis

Specimens were cut sagittally, fixed in 10% formalin for 48 h, and embedded into a paraffin block. Thin-sliced sections (4 *μ*m) were prepared and stained by hematoxylin and eosin (H&E) staining and Masson's trichrome staining by an experienced pathologist who did not know the origins of the specimens. H&E staining revealed the morphology and structure of the LF and the degree of elastin degradation. Masson's trichrome staining was used to identify the elastic fiber (pink) and collagenous fiber (blue) and to determine the degree of fibrosis [16].

### 2.4. Real-Time PCR

Total RNA was isolated from samples or cells by using TRIZOL (Invitrogen Corp., Carlsbad, CA). To measure types I and III collagen mRNA expression levels, cDNA was reverse transcribed from isolated RNA by incubating 500 ng of DNase treated RNA with a first-strand synthesis kit (Advanced Biotechnologies). Real-time PCR was performed using SYBR green dye in a thermal cycler with the following parameters: 40 cycles, 94°C for 30 seconds, 60°C for 20 seconds, and 72°C for 15 seconds. To measure the level of miR-155, RT-PCR was performed with the All-in-One miRNA qRT-PCR Detection Kit (Tiangen). The following primers were used: collagen I forward: 5′-GTGCGATGACGTGATCTGTGA-3′, reverse: 5′-CGGTGGTTTCTTGGTCGGT-3′; collagen III forward: 5′-GCCAAATATGTGTCTGTGACTCA-3′ and reverse: 5′-GGGCGAGTAGGAGCAGTTG-3′; miR-155 forward TTAATGCTAATCGTGATAGGGGT. All primers were synthesized by Shenggong Inc. All data were analyzed using the comparative ΔΔCT method to calculate the difference between the threshold cycle (CT) values of the target and reference genes in each sample.

### 2.5. Western Blot Analysis

Tissue specimens and cultured cells were lysed in RIPA buffer containing protease inhibitor cocktail. Equivalent amounts of protein were separated by electrophoreses and transferred onto an Immobilon-P Transfer Membrane (Millipore). The membranes were blocked with 5% nonfat milk in Tris-buffered saline and then incubated with Col 1 or Col 3 monoclonal antibodies (Abcam, USA), followed by incubation with horseradish peroxidase-conjugated secondary antibody (Abcam, USA). Actin (Abcam, USA) was used as a loading control.

### 2.6. Primary Culture of LF Fibroblast Cells

The LF samples were obtained aseptically from six young patients undergoing spinal surgery. The dissected specimens were minced into small pieces and digested in serum-free medium (Gibco) containing 250 U/mL type I collagenase (Sigma) at 37°C in an atmosphere containing 5% CO_2_. The digested specimens were washed with serum-containing medium to inhibit collagenase activity and then placed in 35 mm dishes in Dulbecco's Modified Eagle Medium and Ham's F-12 medium (DMEM/F12, Gibco) supplemented with 10% heat-inactivated fetal bovine serum (FBS, Gibco-BRL). The cultures were incubated at 37°C in a humidified atmosphere containing 5% CO_2_. The medium was changed every two days. After two weeks, cells began to migrate from the ligament chips and formed a monolayer. The cells were maintained for two to three weeks in DMEM/F12 containing 10% FBS, 1% v/v penicillin, and streptomycin (Sigma) in an incubator with a humidified atmosphere containing 5% CO_2_. The protein expression levels of types I and III collagen were detected by immunocytochemistry as previously described [13].

### 2.7. Vector Construction

hsa-miR-155 precursor sequence was obtained from miRBase (http://www.mirbase.org/): CTGTTAATGCTAATCGTGATAGGGGTTTTTGCCTCCAACTGACTCCTACATATTAGCATTAACAG. hsa-miR-155 sequence was UUAAUGCUAAUUGUGAUAGGGGU, and the complementary DNA sequence was ACCCCTATCACAATTAGCATTAA. The miR-155 sponges were designed as three incomplete complementary sequences. The oligos were cloned into pCDH-GFP to make pCDH-miR-155 or pCDH-miR-155-sponge plasmid.

### 2.8. Lentivirus Packaging and Infection

For transfection, 293T cells were cultured at 37°C in a 5% CO_2_ incubator till the cells reached 70–80% confluence, and the monolayer cells were transfected with PCDH-GFP, pCDH-miR-155, or pCDH-miR-155-sponge plasmid mixed with psPAX2 and pMD using liposomes. The culture medium was discarded after 4 h, and the cells were washed 3 times with PBS. Finally, 15 mL of cell culture medium containing 10% fetal bovine serum was added and the cells were cultured for 24 h. Then, the supernatants containing GFP (blank group), miR-155 (OE group), or miR-155 sponge (Sponge group) lentiviruses were collected and used to infect fibroblast cells. Another lentivirus containing a nonsense miRNA was used as a control (CON group).

### 2.9. Statistical Analysis

Data were expressed as the mean ± SD and analyzed using SPSS version 12 statistical analysis package (SPSS Inc., Chicago, IL, USA). The measurements of LF thickness were compared using student's* t*-test. Differences in collagen I, collagen III, mRNA, and protein expression levels and miR-155 level between groups were evaluated by one-way ANOVA. The correlations among LF thickness, collagen I, collagen III, and miR-155 levels were analyzed using Spearman method. *P* < 0.05 was considered statistically significant.

## 3. Results

### 3.1. Thickness of the LF

The thickness of the middle portion of LF was measured. The mean thickness of LF was 2.8 ± 0.7 mm (range: 1.63–3.87 mm) in LDH group and 5.3 ± 1.0 mm (range: 3.95–7.48 mm) in LSS group. This difference was significant (*P* < 0.01).

### 3.2. Elastin Degradation and Fibrosis of LF

**Figure 1:**
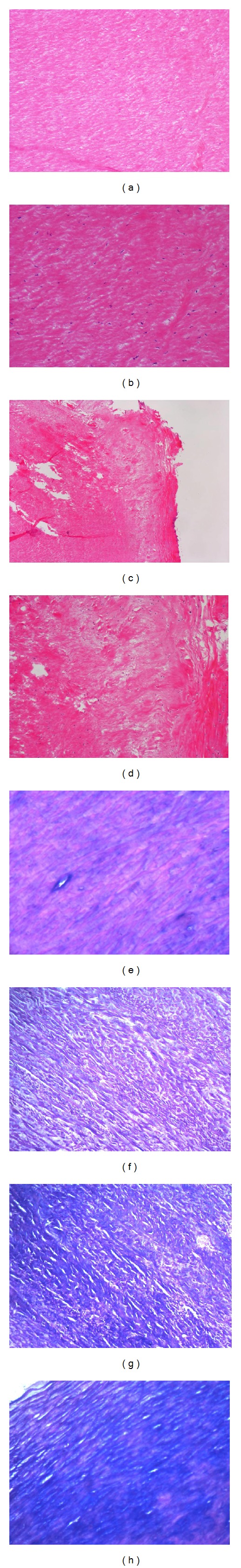
Histological analysis of  LF specimens from LSS and LDH patients. (a) In LF from LDH patients, a large area was stained pink with a regular arrangement, indicating a normal, nonfibrotic condition. H&E staining ×40. (b) LF from LDH patients. H&E staining ×100. (c) In LF from LSS patients, elastic fibers were disorganized and focally lost, accompanied by a proliferation of collagen fibers. The elastic fibers also had low volumes and uneven diameters. H&E staining ×40. (d) LF from LSS patients. H&E staining ×100. (e) In LF from LDH patients, rich elastic fibers were regularly arrayed. In grade 0, <20% area of the LF was stained blue. Masson's trichrome staining ×40. (f) LF from LDH patients. In grade 1, <40% area of the LF was stained blue. Masson's trichrome staining ×40. (g) In LF from LSS patients, a large area was stained blue, indicating the presence of massive fibrosis. In grade 3, over 60% of the entire area was stained blue. Masson's trichrome staining ×40. (h) In LF from LSS patients, in grade 4, over 80% of the entire area was stained blue. Masson's trichrome staining ×40.

Histological analysis showed that the elastic fiber area decreased and collagen area increased in LF from LSS group, compared to LDH group. In LDH group, rich elastic fibers were arrayed in parallel order (Figures 1(a) and 1(b)). However, in LSS group, the elastic fibers were fragmented, disorganized, and focally lost, accompanied by the proliferation of collagen fibers (Figures 1(c) and 1(d)). Masson's trichrome staining showed that, in LF from LDH group, a large area was stained pink and showed a regular arrangement, indicating a normal nonfibrotic condition (Figures 1(e) and 1(f)), but in LF, from LSS group, a large area was stained blue, indicating the presence of massive fibrosis (Figures 1(g) and 1(h)).

### 3.3. Collagens I and III Expression in LF

**Figure 2:**
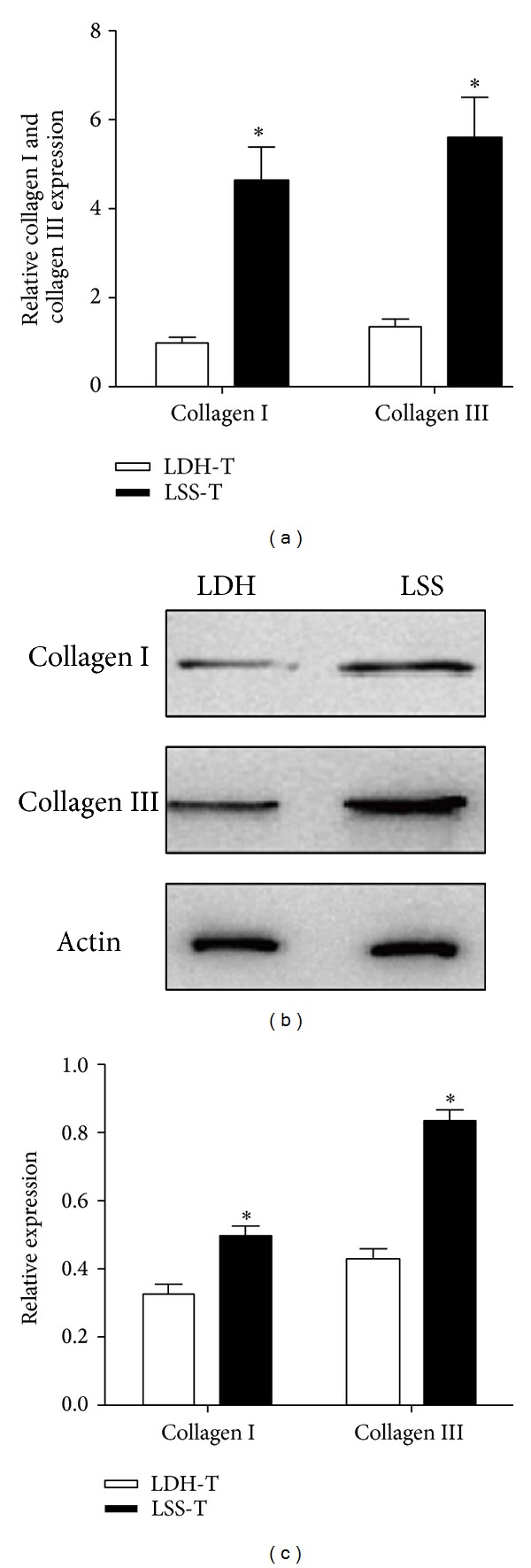
The expression of collagen I and collagen III in LF specimens from LSS and LDH patients. (a) Real-time PCR analysis showed that the relative mRNA levels of collagen I and collagen III in LF were significantly higher in LSS group than in LDH group. (b) Western blot analysis of collagen I and collagen III expression in LF from LSS and LDH groups. (c) The relative protein levels of collagen I and collagen III in LF were significantly higher in LSS group than in LDH group.

The mRNA expression levels of both collagens I and III were increased significantly in LF samples from LSS group (Figure 2(a)). The expression of collagens I and III protein is higher in LF samples from LSS group than from LDH group (Figures 2(b) and 2(c)).

### 3.4. miR-155 Level in LF

**Figure 3:**
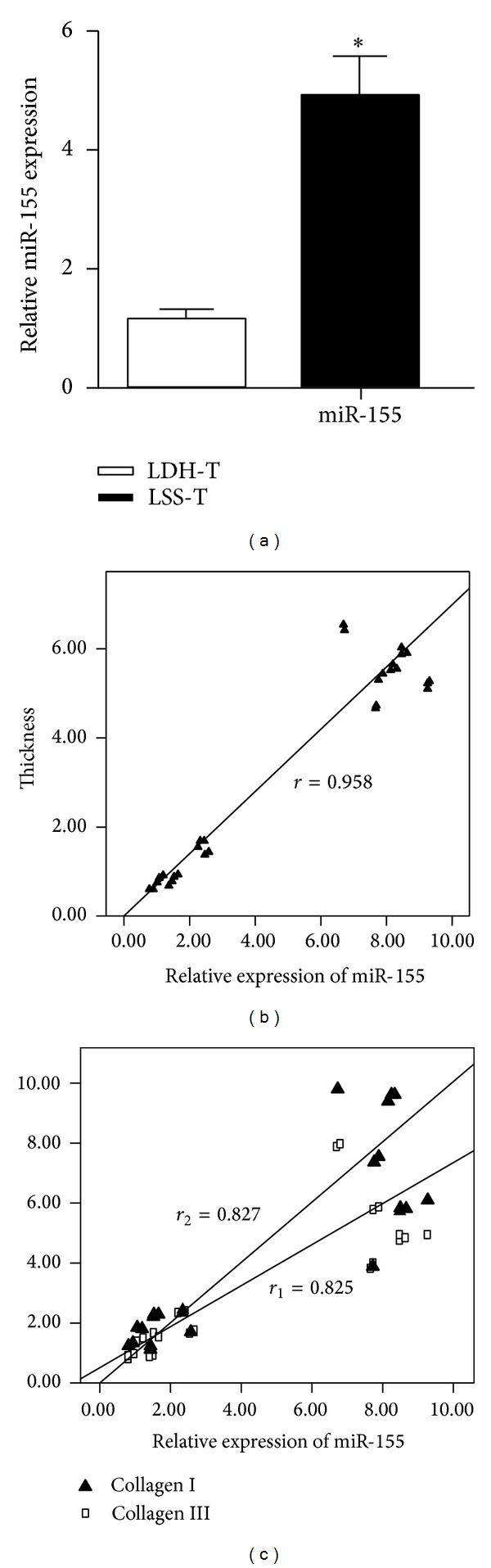
The expression of miR-155 in LF specimens from LSS and LDH patients. (a) The relative miR-155 level in LF was higher in LSS group than in LDH group. (b) Correlation between miR-155 level and LF thickness. (c) A scatter plot showing the positive correlations between miR-155 level and collagen I/III protein levels in LF. The correlation coefficients were 0.825 (*P* < 0.01) and 0.827 (*P* < 0.01), respectively.

The mean level of miR-155 was 1.1748 ± 0.047 (range: 1.1278–1.2218) in LDH group and 5.1081 ± 0.703 (range: 4.4051–5.8111) in LSS group. miR-155 level was significantly higher in LF samples from LSS group than in those from LDH group (*P* < 0.01, Figure 3(a)). Correlation analysis showed that miR-155 level increased in parallel with the thickness of the LF, showing a positive and significant correlation. The regression coefficient was 0.958 (Figure 3(b)). In addition, the expression of types I and III collagen showed a positive and significant correlation with miR-155 level in LF (Figure 3(c)). The regression coefficients were 0.827 (*P* < 0.01) and 0.825 (*P* < 0.01) for types I and III collagen, respectively.

### 3.5. miR-155 Upregulates Collagens I and III Expression in LF Fibroblasts

**Figure 4:**
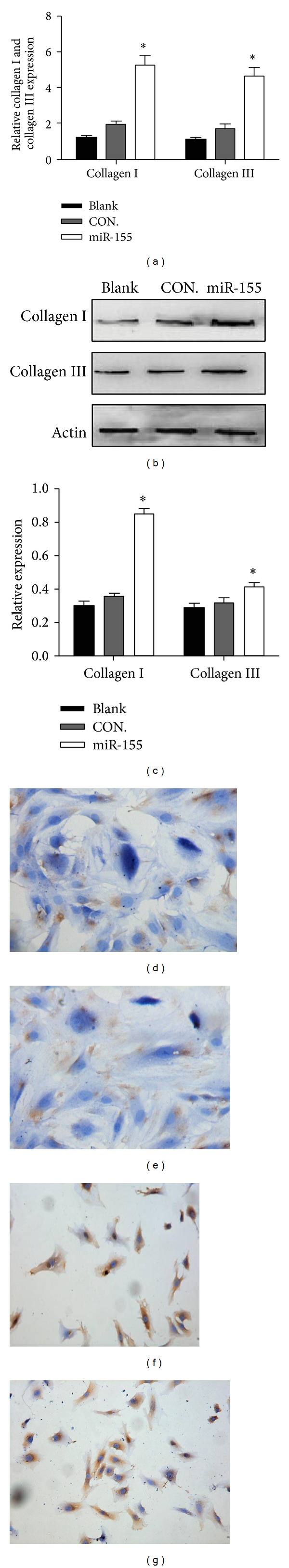
miR-155 mimic increases the expression of collagen I and collagen III in fibroblasts isolated from LF. (a) Real-time PCR analysis showed that the relative mRNA levels of collagen I and collagen III were significantly higher in OE group than in Blank and Control groups. (b) Western blot analysis of collagen I and collagen III expression in different groups of fibroblasts. (c) The relative protein levels of collagen I and collagen III were significantly higher in OE group than in Blank and Control groups. (d) Blank group. (e) Control group. (f) Immunocytochemical staining of collagen I in OE group. (g) Immunocytochemical staining of collagen III in OE group. Brown indicated positive staining.

To determine the role of miR-155 in the regulation of types I and III collagen expression in LF, we isolated fibroblasts from LF and infected them with lentiviruses. RT-PCR analysis showed that mRNA expression of types I and III collagen was increased in cells infected with miR-155 mimic lentivirus compared to controls (Figure 4(a)). Western blot analysis showed that protein expression of types I and III collagen was increased in cells infected with miR-155 mimic lentivirus compared to controls (Figures 4(b) and 4(c)). Immunocytochemistry confirmed that the staining of types I and III collagen was obviously stronger in cells infected with miR-155 mimic lentivirus compared to controls (Figures 4(d)–4(g)).

**Figure 5:**
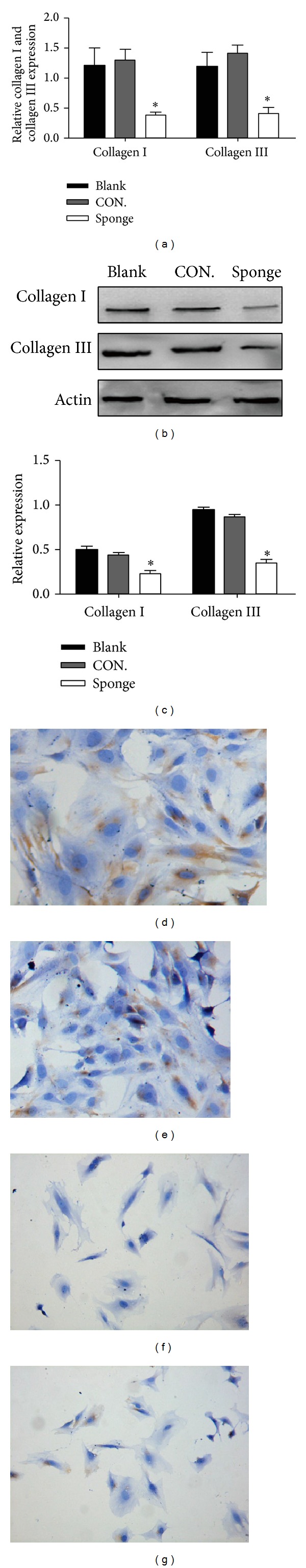
miR-155 sponge decreases the expression of collagen I and collagen III in fibroblasts isolated from LF. (a) Real-time PCR analysis showed that the relative mRNA levels of collagen I and collagen III were significantly lower in Sponge group than in Blank and Control groups. (b) Western blot analysis of collagen I and collagen III expression in different groups of fibroblasts. (c) The relative protein levels of collagen I and collagen III were significantly lower in Sponge group than in Blank and Control groups. (d) Blank group. (e) Control group. (f) Immunocytochemical staining of collagen I in Sponge group. (g) Immunocytochemical staining of collagen III in Sponge group. Brown indicated positive staining.

In contrast, mRNA expression of types I and III collagen was decreased in cells infected with miR-155 sponge lentivirus compared to controls (Figure 5(a)). Consistently, Western blot analysis showed that protein expression of types I and III collagen was decreased in cells infected with miR-155 sponge lentivirus compared to controls (Figures 5(b) and 5(c)). Immunocytochemistry confirmed that the staining of types I and III collagen was weaker in cells infected with miR-155 sponge lentivirus compared to controls (Figures 5(d)–5(g)).

## 4. Discussion

LF contains high concentration of elastin, allowing the contraction during flexion and the elongation during extension [28]. LF hypertrophy is known to cause LSS, leading to low back pain [29]. In this study, we found that mean thickness of LF in LSS group was significantly greater than that in LDH group. We also observed irregularly arranged, ruptured, swollen, and decreased elastic fibers, hyalinization, and increased number of collagen fibers in LF from LSS group. Moreover, we found that LF from LSS group showed a high fibrosis score. These results are consistent with previous studies showing an increase in collagen content (fibrosis) and a decreased elastin-to-collagen ratio in hypertrophied LF [5–7].

To our knowledge, this is the first report describing the relationship between LF hypertrophy and miRNA expression. Recent studies have revealed an important role of miR-155 in the pathogenesis of osteoarthritis and fibrotic diseases [21, 24, 30–32]. Here, we focused on the role of miR-155 in the pathogenesis of LF hypertrophy. We found that miR-155 was significantly upregulated in LF tissue of LSS patients compared with LDH patients. Correlation analysis showed that increased miR-155 expression was correlated with the thickness, fibrotic score, and the expression of types I and III collagen in LF. Furthermore, we isolated fibroblasts from LF and showed that miR-155 upregulated the expression of type I and type III collagen in these cells.

Chronic inflammation is an important risk factor for LF hypertrophy. The progression of LF hypertrophy is accompanied by a high degree of macrophage infiltration [15]. Furthermore, macrophages were identified as a major cellular source of inflammatory cytokines in LF hypertrophy [16]. Recently, miR-155 has been identified as a component of the primary macrophage response to inflammatory mediators [26]. Furthermore, several studies have shown that transforming growth factor-*β* (TGF-*β*) is involved in the process of hypertrophic changes during LF [15, 16]. Interestingly, miR-155 is a direct target of TGF-*β*/Smad pathway, which induces miR-155 expression through Smad4 [33]. miR-155 contributes to the regulation of TGF-*β*/Smad pathway by directly targeting SMAD2 and SMAD5 [34]. These findings establish a strong link between miR-155, TGF-*β* pathway, and LF hypertrophy and indicate that miR-155 is a critical factor that regulates fibroblast fibrosis and LF hypertrophy.

There are several limitations to our present study. First, our sample size was limited by the ethics review board. Second, we could not collect normal LF and instead used control specimens from patients with disc herniations, whose average age was significantly younger than that of the patients with spinal stenosis. Therefore, we can not exclude the possibility that the aging may have an impact on the expression of miR-155. It would have been ideal to have two different controls: age and gender matched specimens from patients without stenosis and gender matched specimens from late-teenagers. The former would eliminate age as an independent variable, since not all old patients develop spinal stenosis. It may be that, much like atherosclerosis, a genetic predisposition alters the biochemical factors that contribute to the pathologic condition. Specimens from late-teenagers who have no any degenerative changes would allow us to determine the extent of the perturbations occurring in spinal stenosis. Unfortunately, both of these ideal controls rarely undergo surgical treatment, making it nearly impossible to gather enough samples in a timely fashion. Thus, we finally settled on our present controls. Although admittedly they are not ideal, they are reasonable and adequate to test the hypothesis we postulated in this study. In fact, in the literatures about ligamentum flavum hypertrophy, young disc herniation group was frequently used as a control group [10].

## 5. Conclusions

In summary, we found that miR-155 was upregulated in patients with lumbar ligamentum flavum hypertrophy. The expression level of miR-155 was correlated with the thickness and the degree of fibrosis of LF. miR-155 increased the expression of types I and III collagen in fibroblasts from LF. These data suggest that miR-155 is a fibrosis-associated miRNA and may play important role in the pathogenesis of LF hypertrophy.
